# Therapeutic Efficacy of Chinese Patent Medicine Containing Pyrite for Fractures: A Systematic Review and Meta-Analysis

**DOI:** 10.3390/medicina60010076

**Published:** 2023-12-30

**Authors:** Eun-Young Nam, Su Hyun Choi, Ji Hye Hwang

**Affiliations:** 1 Mimi Korean Medicine Clinic, Seoul 05616, Republic of Korea; obgyey@gmail.com; 2 College of Korean Medicine, Gachon University, Seongnam 13120, Republic of Korea; youngeun0922@naver.com; 3 Department of Acupuncture & Moxibustion Medicine, College of Korean Medicine, Gachon University, Seongnam 13120, Republic of Korea

**Keywords:** Chinese patent medicine, fracture, pyrite, pyritum, systematic review, meta-analysis

## Abstract

*Background and Objectives*: Korean and traditional Chinese medicine state that pyrite is effective for fracture treatment, but supporting clinical data are limited. This systematic review aimed to investigate the therapeutic role of Chinese patent medicine containing pyrite (CPMP) in clinical treatment for fractures. *Materials and Methods*: Seven electronic databases were searched using the keywords “pyrite”, “pyritum”, and “zirantong” between inception and December 2022, yielding 29 published clinical studies. Randomized controlled trials that included CPMP were considered eligible regardless of the fracture type. Quality assessment and meta-analysis of the included RCTs were also performed. *Results*: Most studies showed high heterogeneity (I^2^ > 50%) and significant results (*p* < 0.05). Compared to the results of the control group, CPMP was more effective in terms of the primary outcome related to the efficacy rate, including the total effective rate, callus growth rate, bone union, and edema disappearance time (all *p* < 0.00001) and in terms of secondary outcomes related to pain reduction, namely pain intensity and pain disappearance time, than the control group (both *p* < 0.01). CPMP was more effective than the control group in terms of erythrocyte sedimentation rate (*p* < 0.01), hematocrit (*p* < 0.01), erythrocyte aggregation (*p* < 0.05), and plasma viscosity (*p* < 0.05). CPMP did not cause serious side effects, and the incidence of complications was significantly less than that in the control group. *Conclusions*: CPMP may be a safe and effective alternative treatment for fractures and may be beneficial in preventing postoperative complications, reducing pain, relieving symptoms, and accelerating healing.

## 1. Introduction

Bone is a complex mineralized connective tissue arranged in a multi-scale hierarchical configuration, and there is a strong dependence between the mechanical properties of bone and its meso- and microstructural arrangement [1]. This complexity of bone is also being exploited for torsion-resistant bio-inspired solutions in the medical field [2]. Bone has stress-concentrating features such as natural voids and defects, as well as voids caused by pathological conditions or created during surgery. These features reduce the mechanical integrity of the bone, making it more susceptible to sudden brittle fracture during trauma or gradual fatigue failure over time. Large defects caused by disease and surgery can create holes, notches, sharp edges, and cracks [3]. In severe fractures, hemorrhagic shock due to blood vessel damage can be life threatening, and in some cases, may damage the internal organs [4,5]. In 2019, 178 million new fractures were reported worldwide, representing a 33.4% increase since 1990 [6]. Fractures are a global public health challenge that can lead to a high burden on individuals, families, societies, and healthcare systems through reduced productivity, disability, reduced quality of life, and high healthcare costs [7,8,9].

A common fracture treatment approach is fixing the broken bone with a cast and administering anti-inflammatory drugs to relieve pain and inflammation [10]. However, anti-inflammatory drugs greatly decrease fracture treatment efficiency by inhibiting COX-1 and COX-2 functions, which play important roles in the early recovery from fractures [11]; thus, their use has been avoided. It is recommended to take an overdose of calcium in addition to vitamin D, which acts as a calcium absorption supplement, although the overdose can cause various side effects such as vomiting, diarrhea, and convulsions [12]. The most commonly used drugs include bisphosphonates, teriparatide, and parathyroid hormone; however they can cause serious side effects, such as jawbone necrosis, dyspnea, tissue damage, gastroesophageal reflux disease, rash, joint pain, and headache [13]. Movement restriction due to the long duration associated with t fracture treatment can cause pneumonia, deep vein thrombosis, reduced maintenance of physical function, and quality of life [14,15]. Therefore, it is crucial to develop an economical fracture treatment agent that promotes bone formation, has few side effects, and is easy to administer [16,17].

Recently, market interest has shifted from developing single and complex compounds with various side effects to novel natural drugs and product extracts with relatively low side effects and multiple mechanisms of action [18,19]. The Republic of Korea, China, and Japan have a long history of the clinical use of natural products with well-established systematic records and traditional theories [19]. Pyrite has been used in traditional mineral medicine in the Republic of Korea and China for thousands of years. It eliminates blood stasis and connects muscles and bones [20] and has been widely used in fracture treatment [21]. In Korean medicine (KM), pyrite is often used alone or as a component of combination treatment in clinical practice; however, there have been few clinical studies examining its effect [22]. In traditional Chinese medicine (TCM), several clinical studies using pyrite have been reported. Particularly, Chinese patent medicine (CPM) containing pyrite (CPMP) has been used for fracture treatment [23,24] although a comparative analysis of CPMP efficacy in fracture treatment is lacking. Therefore, this study systematically reviewed the currently available literature and performed a meta-analysis regarding CPMP efficacy and safety in clinical practice.

## 2. Materials and Methods

The protocol for this systematic review was previously published [25] and registered in the International Prospective Register of Systematic Reviews (PROSPERO). This systematic review was reported in compliance with the Preferred Items for Systematic Reviews and Meta-Analyses (PRISMA) [26].

### 2.1. Data Sources and Search Strategy

Seven electronic databases, namely EMBASE, PubMed, Cochrane Central Register of Controlled Trials, China National Knowledge Infrastructure (CNKI), Korean Studies Information Service System (KISS), National Digital Science Library (NDSL), and Oriental Medicine Advanced Searching Integrated System (OASIS), were searched for published literature from their inception to May 2023. RCTs that included CPMP were considered eligible regardless of fracture type. Language restrictions were not imposed. The keywords “pyrite”, “pyritum”, and “zirantong” were used for the search. The CPMP search was conducted in CNKI using CPMPs contained in the Chinese Pharmaceutical Dictionary and the Newly Edited National Chinese Traditional Patent Drugs.

### 2.2. Inclusion and Exclusion Criteria

#### 2.2.1. Study Types

Prospective RCTs evaluating CPMP effectiveness for bone fractures were included. Other types of studies, such as non-RCTs, case reports, review articles, and animal experiments, were excluded. The exclusion criteria were studies (1) reporting diseases not related to fractures; (2) reporting unclear outcomes; (3) without outcome information; (4) not eligible for outcome measurements; and (5) duplicates. Studies of parenteral administration, such as the external use of CPMP, were excluded.

#### 2.2.2. Participant Types

Patients diagnosed with fractures by radiographic examination or who met the Chinese standard fracture diagnostic criteria [27] were included in the study regardless of fracture sites and types. There were no restrictions on patient age, sex, or nationality.

#### 2.2.3. Intervention and Control Types

The concomitant administration of CPMP and application of conventional treatments, such as reduction, fixation, and Western medication, were analyzed as an intervention group; however, there were no specific criteria for CPMP administration type or duration. The control group was set as having administration of either conventional treatments or Western medication. The patients of both the intervention and control groups should have received the same conventional treatment.

#### 2.2.4. Outcome Measurement Types

The measured primary outcome was the efficacy rate, including the total effective rate, callus growth rate, bone union, and edema disappearance time.

The assessed secondary outcomes were pain reduction—using the visual analog scale (VAS)—and pain disappearance time. The erythrocyte sedimentation rate (ESR), hematocrit (Hct), erythrocyte aggregation (EA), and plasma viscosity (PV) were evaluated using the blood test results.

### 2.3. Data Extraction

Identified articles were managed using Endnote software (version 20) (Clarivate, London, UK) for inclusion eligibility and screened based on the inclusion and exclusion criteria; duplicate articles were manually removed. For the selected studies, two researchers (anonymized for review) independently extracted the following data using a predefined MS Excel (Microsoft Office 2016, Microsoft Corp., Redmond, WA, USA) format: author, year of publication, study design, participants characteristics (age and sex), sample size, diagnostic criteria of fracture, classification of diseases, detailed information on the intervention and control treatments (method, duration, and dosage), main outcome measurements, results, adverse effects, and blinding method. Disagreements or uncertainties between researchers were resolved by discussion with a third reviewer (anonymized for review).

### 2.4. Data Analyses

After extracting the necessary data from the selected literature, a meta-analysis was conducted using Cochrane Review Manager 5.4.1 software (The Nordic Cochrane Centre, The Cochrane Collaboration, Copenhagen, Denmark). The corresponding authors of the studies with missing information were contacted whenever possible to acquire and verify the data. When appropriate, data across the studies were pooled to conduct a meta-analysis using fixed or random effects. GRADEpro software (https://www.gradepro.org/, accessed on 1 January 2023) from the Cochrane Systematic Reviews was used to create tables with a summary of findings. If the necessary data were available, a subgroup analysis was performed for the different types of therapies available and compared with CPMP. The total effective rate and callus growth rate are presented as odds ratios (OR) and 95% confidence intervals (CI), while the continuous variables are presented as standardized mean difference (SMD) and 95% CI. Statistical heterogeneity was assessed by I^2^ and *p*-values based on 95% CIs, where I^2^ < 25% was considered low heterogeneity; I^2^ = 25–50% was considered moderate heterogeneity; I^2^ > 50% was considered high heterogeneity; and *p* < 0.05 was considered statistically significant. The meta-analysis used random-effects or fixed-effects models if the heterogeneity was significant or insignificant, respectively. A subgroup analysis was performed using the X2 test, and sensitivity analysis was conducted to determine the robustness of the merged results by deleting low-quality studies. Publication bias was evaluated using funnel plots.

### 2.5. Quality Assessment

The RCTs quality was evaluated using the Cochrane Risk of Bias tool version 5.1.0 with the following aspects: random sequence generation, allocation concealment, blinding of patients and personnel, incomplete outcome data, selective reporting, and other types of bias. The assessment results were classified using “L”, “U”, and “H” to indicate low, uncertain, and high risk of bias, respectively. Quality assessment was independently performed by two researchers. If there were disagreements in the evaluations between the two researchers, the literature was rechecked. If a disagreement persisted, an agreement was reached through discussion with a third party. If information could not be obtained from an article for assessment, the corresponding author was contacted.

### 2.6. Ethics and Dissemination

Ethical approval and patient consent were not required because this meta-analysis was based on published research results.

## 3. Results

### 3.1. Literature Search

**Figure 1:**
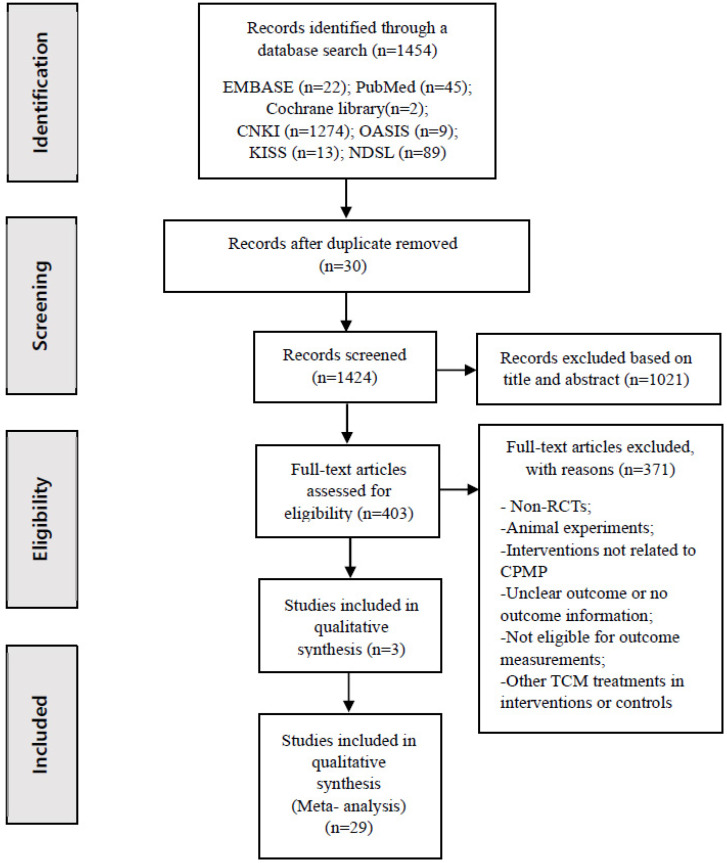
PRISMA flow chart diagram. CNKI, China National Knowledge Infrastructure; OASIS, Oriental Medicine Advanced Searching Integrated System; KISS, Korean Studies Information Service; NDSL, National Digital Science Library; RCTs, randomized controlled trials; CPMP, Chinese patent medicines containing pyritum, TCM, traditional Chinese medicine.

The procedure for the literature search and screening is presented in Figure 1. Only pyrite-related studies, such as TCM or traditional herbal medicines, were selected for review from the relevant literature. A total of 1454 articles were identified, including 22 from EMBASE, 45 from PubMed, 2 from Cochrane, 1274 from CNKI, 9 from OASIS, 13 from KISS, and 89 from NDSL. Among them, 30 duplicate studies were excluded. After screening the titles and abstracts, 1021 studies that were not related to fractures or CPMP were excluded. The full texts of the 403 remaining articles were assessed for eligibility. In total, 371 articles were excluded for the following reasons: non-RCTs (such as case reports or review articles), animal experiments, unclear outcomes, no outcome information, ineligibility for outcome measurements, and other Chinese treatments in the intervention or control groups. Among the 32 selected studies, three studies were excluded because they did not present the results of the total effective rate, VAS, pain reduction, blood test, and other related results. Finally, 29 studies were included in this systematic review.

### 3.2. Study Characteristics

The characteristics of the 29 included studies are presented in Table 1 and Table 2. Among the data of the 3206 collected patients, there were 1612 and 1594 patients in the treatment and control groups, respectively. They were aged 7–85 years; the age varied according to the location and etiology of the fracture. Diagnostic criteria were based on Chinese fracture diagnosis criteria. Most studies were conducted after undergoing surgery or administrating primary treatment. Although the diagnostic criteria differ slightly according to location, most were based on imaging and morphology. Table 3 summarizes the evidence and main effects of CPMP therapy on fractures.

### 3.3. CPMPs Used in the Treatment Groups

Seven CPMPs, namely Diedashenggukeli [28], Guyulingjiaonang [29,30,31], Guzhecuoshangjiaonang [32], Huoxuezhitong [33,34,35,36], Jieguwan [37,38], Sanhuajiegusan [39,40], and Shangkejiegupian [41,42,43,44,45,46,47,48,49,50,51,52,53,54,55,56], were used in the treatment groups. Among them, Shangkejiegupian, which consists of 12 ingredients, was used by 2101 patients (65.35%) in 16 studies. Huoxuezhitong, with six ingredients, was used by 406 patients (12.63%) in four studies. Jieguwan, with nine ingredients, was used by 214 patients (6.66%) in two studies. Sanhuajiegusan, with 18 ingredients, was used by 196 patients (6.10%) in two studies. Guyulingjiaonang, with 16 ingredients, was used by 173 patients (5.38%) in three studies. Diedashenggukeli, with eight ingredients, was used by 80 patients (2.49%) in one study. Guzhecuoshangjiaonang, with 12 ingredients, was used by 45 patients (1.40%) in one study.

**Figure 2:**
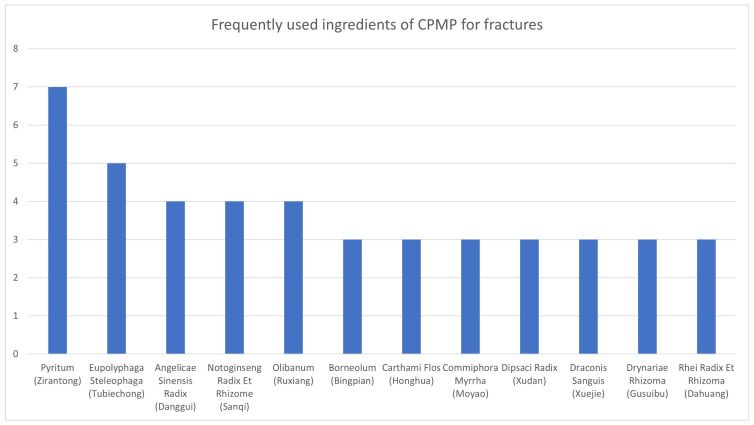
Frequently used ingredients in Chinese patent medicine containing pyrite (CPMP) for fractures.

The 41 constituents included in all CPMPs are listed in Table 4. Among medicinal raw materials, along with pyrite, *Eupolyphaga steleophaga* (Tubiechong), Angelicae Sinensis Radix (Danggui), Notoginseng Radix Et Rhizome (Sanqi), and Olibanum (*Ruxiang*) were found to have the highest frequency for combined use (Figure 2). Pyrite was included in all CPMPs. *Eupolyphaga steleophaga* (Tubiechong) was included in five CPMP types; Angelicae Sinensis Radix (Danggui), Notoginseng Radix Et Rhizome (Sanqi), and Olibanum (*Ruxiang*) were included in four CPMP types; *Borneolum* (Bingpian), *Carthami flos* (Honghua), *Commiphora myrrha* (Moyao), Dipsaci Radix (Xudan), *Sanguis draconis* (Xuejie), Drynariae Rhizoma (Gusuibu), and Rhei Radix Et Rhizoma (Dahuang) were included in three CPMP types; and Achyranthis Bidentatae Radix (Niuxi), Melo Semen (Tianguazi) Pheretima (Dilong), Rhizoma Chuanxiong (Chuanxiong), and Semen Strychni Pulveratum (Maqianzifen) were included in two CPMP types. The other 24 raw materials were included in one type of CPMP each.

### 3.4. Quality Assessment

**Figure 3:**
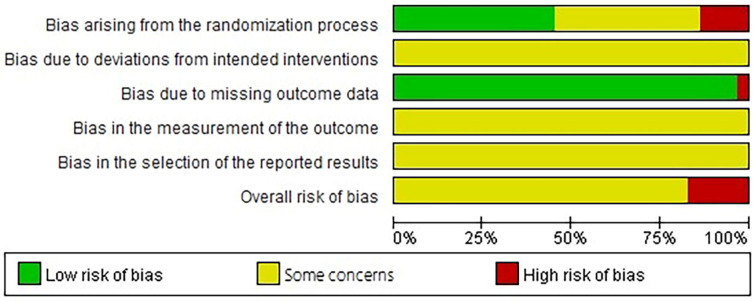
Risk of Bias 2.0 summary: authors’ judgements for each risk of bias domain across all included studies.

The methodological quality of the 29 included studies is summarized in Figure 3 and Table 5. The risk of bias in studies was assessed using the Risk of Bias 2.0 tool [57]. The overall risk of bias, in one or more domains, was evaluated to be of “some concern” in 25 studies and “high” in four studies. In all other studies, the overall risk of bias was evaluated as “high”. Regarding reporting detailed information about the randomization process, 13 studies were assessed as “low” risk, 12 studies as “unclear”, and the remaining 4 studies as “high” risk. Allocation concealment assessed 16 studies as “high” risk and 13 studies as “unclear” risk. Blinding and selective response assessments of all the studies were assessed as “unclear” risks owing to the uncertainty of the information. In the incomplete outcome data and other biases, most studies were assessed as “low” risk, except for one study, which had incomplete outcome data.

### 3.5. Outcomes (Primary Outcomes: The Efficacy Rate)

#### 3.5.1. Total Effective Rate

**Figure 4:**
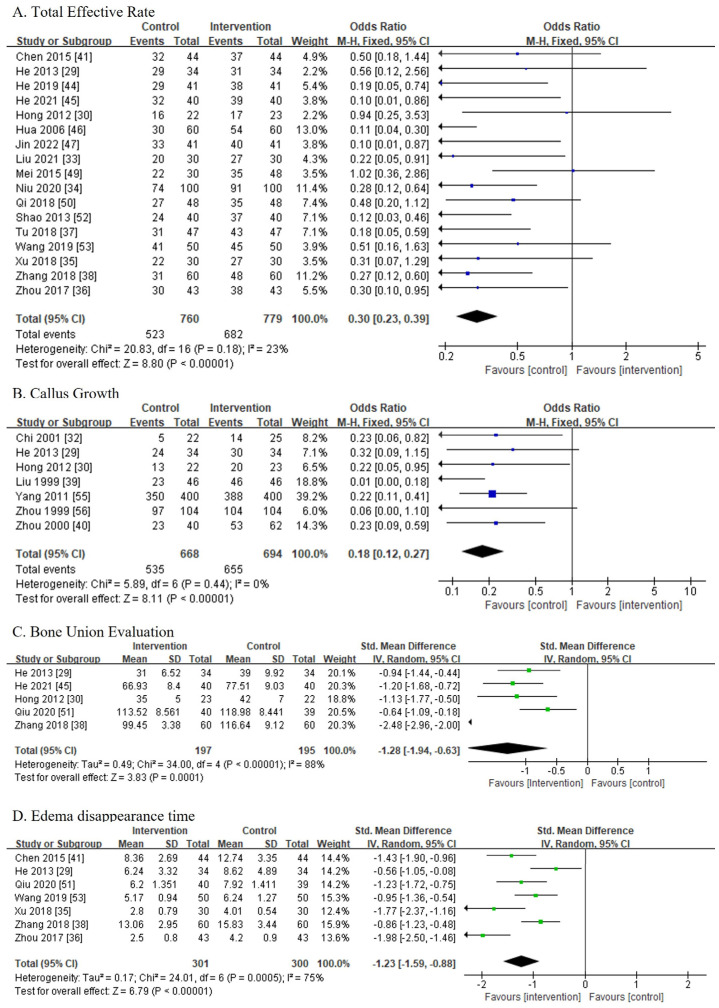
Primary outcome [29,30,32,33,34,35,36,37,38,39,40,41,44,45,46,47,49,50,51,52,53,55,56]. (**A**) Total effective rate. (**B**) Callus growth rate. (**C**) Bone union evaluation. (**D**) Edema disappearance time.

Total effectiveness was reported in 17 studies [29,30,33,34,35,36,37,38,41,44,45,46,47,49,50,52,53] involving 1539 patients. Guyuling [29,30], Huoxuezhitong [33,34,35,36], Jieguwan [37,38], and Shangkejiegupian [41,44,45,46,47,49,50,52,53] were used for analysis. Some studies used Western medication in both the intervention and control groups after surgery, such as vitamin D, Calcium [29], sodium chloride injection with cefazolin sodium pentahydrate [35], cefoxitin sodium with saline [36], eparin sodium injection [47], sodium aescinate [49], and diclofenac sodium injection [53]. As a result, 682 of the 779 patients in the CPMP groups and 523 of the 760 patients in the control groups were effectively treated. Owing to the low heterogeneity among the trials (I^2^ = 23%), a fixed-effects model was chosen for the meta-analysis. The CPMPs were superior to the control groups in terms of increased efficacy (OR, 0.30; 95% CI, 0.23–0.39; *p* < 0.001) (Figure 4A).

#### 3.5.2. Callus Growth Rate

Seven studies [29,30,32,39,40,55,56] involving 1362 patients reported callus growth rates. The intervention groups used Guyuling [29,30], Guzhecuoshang [32], Sanhuajiegusan [39,40], or Shangkejiegupian52,53 to treat CPMP after surgery. Among the seven articles, two used vitamin D, calcium, calcitriol [29], oyster shell calcium and vitamin C [40] in both groups, and one study used benorilate [55] in the control group only. The remaining studies did not indicate the use of the drugs in the control group. A total of 655 out of the 694 patients in the CPMP groups and 535 out of the 668 patients in the control groups were effectively treated. Due to the absence of heterogeneity among the trials (I^2^ = 0%), a fixed-effects model was chosen for the meta-analysis. CPMP treatments were more effective in increasing the callus growth rate than the control treatments (OR, 0.18; 95% CI, 0.12–0.27; *p* < 0.001) (Figure 4B).

#### 3.5.3. Bone Union Evaluation

Five studies [29,30,38,45,51] involving 392 patients reported bone union evaluation; among them, 197 and 195 were included in the CPMP and control groups, respectively. The CPMP groups used Guyuling [29,30], Jieguwan [38], and Shangkejiegupian [45,51] postoperatively. Except for one article that used vitamin D, calcium, and calcitriol [26] in both groups, the other articles did not indicate the use of a drug in the control groups. Owing to the high heterogeneity among the trials (I^2^ = 88%), a random-effects model was chosen for the meta-analysis. The CPMP treatments were more effective for bone union than in the control group treatments (SMD, −1.28, 95% CI, [−1.94, −0.63]; *p* < 0.001) (Figure 4C).

#### 3.5.4. Edema Disappearance Time

Edema disappearance time was reported in seven studies [29,35,36,38,41,51,53] involving 601 patients. There were 301 and 300 patients in the CPMP and control groups, respectively. The CPMP groups used Guyuling [29], Huoxuezhitong [35,36], Jieguwan [38], and Shangkejiegupian [41,51,53], postoperatively. Among them, vitamin D, calcium, and calcitriol [29], sodium chloride injection with cefazolin sodium pentahydrate [35], cefoxitin sodium with saline [36], and diclofenac sodium injection [53] were used in both groups; the other studies did not indicate the use of a drug in the control groups. Owing to the high heterogeneity among the trials (I^2^ = 75%), a random-effects model was chosen for the meta-analysis. The edema disappearance time of the CPMP treatment groups was significantly shorter than that of the control groups (SMD, −1.23; 95% CI, [−1.59, −0.88]; *p* < 0.001) (Figure 4D).

### 3.6. Outcomes (Secondary Outcomes: Pain Reduction)

#### 3.6.1. VAS

**Figure 5:**
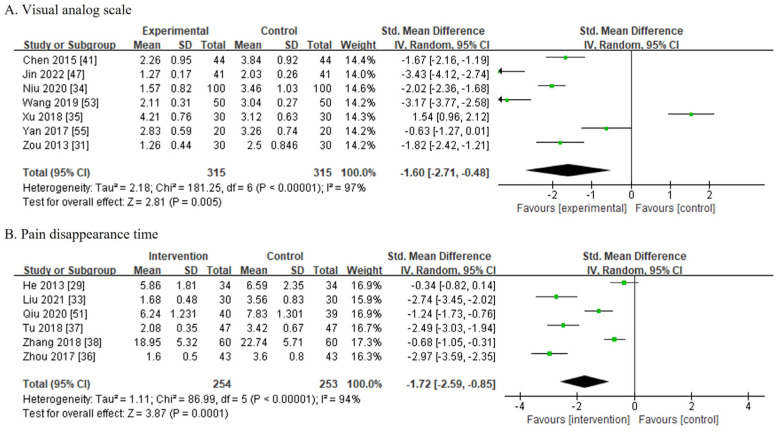
Secondary outcomes (pain reduction) [29,31,33,34,35,36,37,38,41,47,51,53,55]. (**A**) Visual analog scale (VAS) pain scores. (**B**) Pain disappearance time.

Seven studies involving 630 patients reported the VAS score, which were divided into 315 patients from the CPMP groups and 315 patients from the control groups. The CPMP groups used Guyuling [31], Huoxuezhitong [33,34], and Shangkejiegupian [41,47,53,54] postoperatively. Among them, sodium chloride injection with cefazolin sodium pentahydrate [35], heparin sodium injection [47], and diclofenac sodium injection [53] were used in both groups, whereas the other studies did not indicate the use of a drug in the control groups. Owing to the high heterogeneity among the trials (I^2^ = 97%), a random-effects model was chosen for the meta-analysis. The difference of VAS score before and after treatment of the CPMP treatment groups was significantly higher than that of the control groups (SMD, −1.60; 95% CI, [−2.71, −0.48]; *p* = 0.005) (Figure 5A).

#### 3.6.2. Pain Disappearance Time

Six studies [29,33,36,37,38,51] involving 507 patients reported the pain disappearance time. The CPMP and control groups included 254 and 253 patients, respectively. The CPMP groups used Guyuling [29], Huoxuezhitong [33,36], Jieguwan [37,38], and Shangkejiegupian [51] postoperatively. Among them, vitamin D, calcium, and calcitriol [29], mezlocillin injection [33], and cefoxitin sodium with saline [36] were used in both groups, whereas the other studies did not indicate the use of a drug in the control groups. Owing to the high heterogeneity among the trials (I^2^ = 94%), a random-effects model was chosen for the meta-analysis. The pain disappearance time in the CPMP treatment groups was significantly shorter than that in the control groups (SMD, −1.72; 95% CI, [−2.59, −0.85]; *p* = 0.001; Figure 5B).

### 3.7. Outcomes (Others: Blood Test Results)

#### 3.7.1. ESR

**Figure 6:**
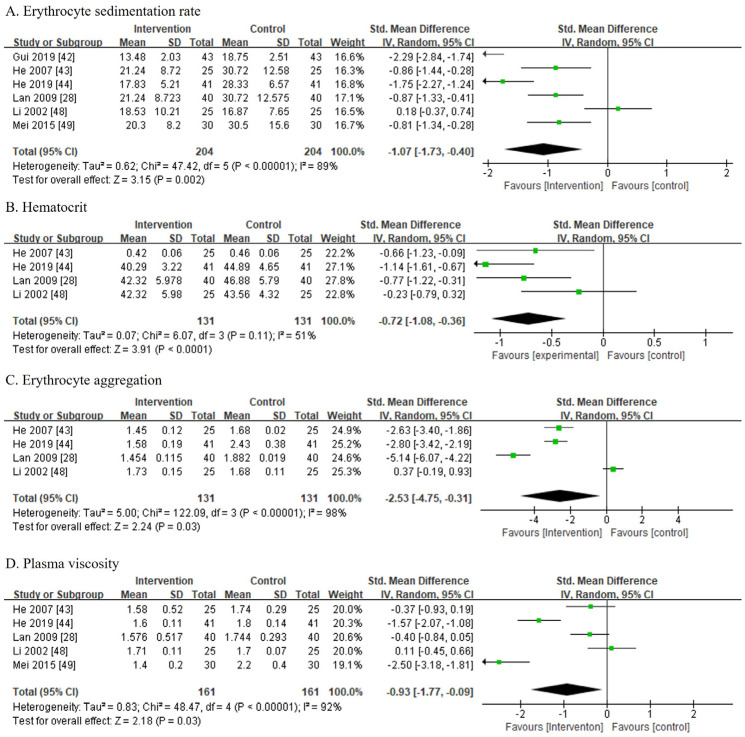
Others (blood test results) [28,42,43,44,48,49]. (**A**) Erythrocyte sedimentation rate. (**B**) Hematocrit. (**C**) Erythrocyte aggregation. (**D**) Plasma viscosity.

Six studies [28,42,43,44,48,49] involving 408 patients reported ESR. There were 204 patients from the CPMP groups and 204 patients from the control groups. The CPMP groups received Diedashenggukeli [28] and Shangkejiegupian [42,43,44,48,49] postoperatively. Among them, sodium aescinate [49] was used in both groups, except for an article that used antibiotics and heparin sodium injection [42] in both groups. The other studies did not indicate the use of a drug in the control groups. Owing to the high heterogeneity among the trials (I^2^ = 89%), a random-effects model was chosen for the meta-analysis. The ESR of the CPMP treatments was significantly lower than that of the control group treatments (SMD, −1.07; 95% CI, [−1.73, −0.40]; *p* = 0.002; Figure 6A).

#### 3.7.2. Hct

Four studies [28,43,44,48] involving 262 patients reported Hct. There were 131 patients from the CPMP groups and 131 patients from the control groups. The CPMP groups used Diedashenggukeli [28] and Shangkejiegupian [43,44,48] postoperatively, while the use of a drug was not indicated in the control groups. The meta-analysis used the SMD and random-effects model owing to the high heterogeneity among the trials (I^2^ = 51%). The Hct of the CPMP treatment groups was significantly lower than that of the control groups (SMD, −0.72; 95% CI, [−1.08, −0.36]; *p* < 0.001; Figure 6B).

#### 3.7.3. EA

Four studies [28,43,44,48] involving 262 patients reported EA. The CPMP and the control groups each comprised 131 patients. The CPMP groups used Diedashenggukeli [28] and Shangkejiegupian [43,44,48] postoperatively, while the use of a drug was not indicated in the control groups. The meta-analysis used the SMD and random-effects model, owing to the high heterogeneity among the trials (I^2^ = 98%). The EA in the CPMP treatment groups was significantly lower than that in the control groups (SMD, −2.53; 95% CI, [−4.75, −0.31]; *p* = 0.03) Figure 6C).

#### 3.7.4. PV

PV was reported in five studies [28,43,44,48,49] involving 322 patients. There were 161 patients from the CPMP groups and 161 patients from the control groups. The CPMP used Diedashenggukeli [28] and Shangkejiegupian [43,44,48,49] postoperatively. Sodium aescinate [49] was used in both groups, whereas the others did not indicate the use of a drug in the control groups. Owing to the high heterogeneity among the trials (I^2^ = 92%), a random-effects model was chosen for the meta-analysis. The PV of the CPMP treatment groups was significantly lower than that of the control groups (SMD, −0.93; 95% CI, [−1.77, −0.09]; *p* = 0.03; Figure 6D).

### 3.8. Safety Assessment

Nine RCTs mentioned adverse effects or complications; among them, four RCTs reported the complications or adverse effects. In Zhou’s study [36], rash occurred in the control group after the treatment administration, and there were no adverse effects in the CPMP group. Zhang and Zhong [38] reported complications that occurred after the administration of fracture treatment. In the CPMP groups, five complications were reported, including one case of fracture displacement, one case of delayed union, one case of dysfunction, and two cases of limb deformity. In the control groups, 16 complications were reported, including fracture displacement in three patients, delayed union in five patients, infection in two patients, dysfunction in three patients, and limb deformity in three patients. There was a statistically significant difference between the CPMP and control groups. He et al. [45] reported one case of deep vein thrombosis in the CPMP group; moreover, they reported one case of infection, three cases of deep vein thrombosis, and two cases of hematoma in the control group, which showed a statistically significant difference. Qiu et al. [51] reported 23 cases of fever and 20 cases of pain at the fracture site after the administration of fracture treatment in the CPMP group, and 23 cases of fever and 19 cases of pain in the control group. In summary, the total rate of complications or adverse effects was 62.5% for the CPMP groups and 61.54% for the control groups, with no statistical significance between the two groups. In addition, five patients experienced complications (two cases of delayed union and three cases of loosening of the internal fixture) in the CPMP groups, and 10 patients experienced complications (five cases of delayed union and five cases of loosening of the internal fixture) in the control groups. The incidences of complications in the CPMP and control groups were 12.5% and 25.64%, respectively, showing a statistically significant difference.

### 3.9. Bias Analysis

**Figure 7:**
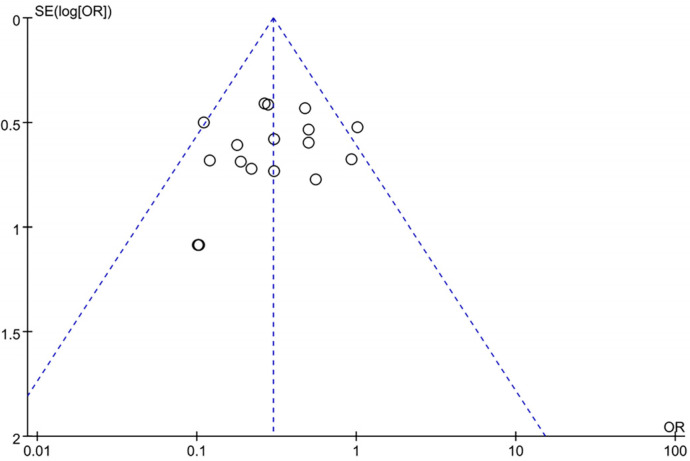
Funnel plots.

Publication bias was assessed using a funnel plot, which was evaluated for the total effective rate reported in 17 studies. A significant symmetry was observed for the distribution in the funnel plots (Figure 7).

### 3.10. Summary of Evidence According to Outcome Measures

For all outcome measures in the CPMP compared with the control trials, the overall quality of the evidence ranged from very low to moderate. Table 6 presents the GRADE assessment results.

## 4. Discussion

Fracture healing is a complex process involving various factors at the cellular and molecular levels, and various mechanisms involving the vasculature, lymphatic vessels, immune cells, polyadenylation, etc., have been reported. The vasculature and lymphatics have been reported to induce bone formation and hematopoietic regeneration [58,59], immune cells have been reported to play a pivotal role in fracture healing in bone, the unique tissue that constitutes the osteoimmune system [60,61], and the field of osteoimmunology continues to uncover new dimensions of the mutual interactions between bone and the immune system [60,62]. In addition, the role of alternative polyadenylation as a post-transcriptional regulatory mechanism involved in transcriptome formation in fracture healing and regulation of gene expression during bone regeneration has recently been reported. During the fracture healing process, it can lead to various complications, such as delayed healing or non-union healing pattern [63]. These complications are difficult to treat and cause a financial burden due to loss of productivity [64]. Accordingly, any strategy that helps reduce the healing time aids in the rapid resumption of the work duties and daily activities by the patients, improvement of their medical outcomes, and reduction of their financial burden. Traditional medicines have been widely used to treat bone diseases for several centuries. According to TKM and TCM theories, the pathological symptoms of fractures are accompanied by redness, pain, and swelling at the fracture site, which may cause circulatory congestion or delay treatment [65]. Studies examining TKM and TCM suggest that herbal medicines can improve fracture healing by controlling inflammation, promoting blood circulation, and stimulating bone formation [66].

### 4.1. Main Findings

This review aimed to establish evidence for the efficacy and safety of CPMP treatment of fractures. Twenty-nine RCTs, with a total of 3206 patients, were included. This study indicated CPMPs had higher therapeutic efficacy by significantly improving the callus growth rate, bone union, and edema disappearance time compared with the control groups receiving no treatment or Western medicine. Moreover, CPMP reduced the pain intensity and duration and had fewer complications without posing serious risks.

In fracture healing, the bone vasculature, a major component of the bone marrow microenvironment, plays a fundamental role in coordinating osteogenesis and hematopoiesis through the production of various angiocrine factors. The vasculature provides signals for the maintenance and proliferation of bone hematopoietic stem and progenitor cells and regulates the differentiation of perivascular mesenchymal stem cells to generate bone cells [58,59]. Our review revealed that all parameters of the blood tests, namely ESR, PV, Hct, and EA, related to changes in the blood circulation and hematoma during fracture healing, were significantly improved in the CPMP groups compared with the control groups. These results suggest that CPMP treatment has an effect on the vascular system, which plays an important role in the fracture healing process.

### 4.2. CPMP Therapeutic Efficacy for Hematomas, a Critical Parameter in Fractures

In general, when a bone fracture occurs, the bone itself and the blood from the damage-induced bleeding in the connective tissue around the fracture coagulate, forming a hematoma. The hematoma in these fractured areas plays a role in biomechanically fixing the fracture area and biologically creating the outer structure of the tissue necessary for cell infiltration and vascular ingrowth. Therefore, many studies have reported that hematomas play an important role in bone fracture healing [67,68], and studies on promoting the fracture healing process have reported that it is important to rapidly improve the blood flow in the fractured area during the fracture healing process [69].

Pyritum is a mineral that is used to treat fractures in KM and TCM. Mineral medicines have been used for more than 4000 years. There are 82 and 34 types of mineral medicines out of 5676 and 514 types of medicines in the *Dictionary of Traditional Chinese Medicine* and *The Korean Herbal Pharmacopeia*, respectively. In China, mineral medicines are recognized as equally important to animal- and vegetable-derived medicines, and research has been conducted on these medicines using modern scientific methods since the late 1970s. However, in the Republic of Korea, there is a lack of systematic mineral herbal medicine research, and some mineral medicines contain heavy metals, such as mercury, arsenic, lead, and copper, which limits their use and results in the scarcity of literature in that respect [70].

In China, a CPM formulation is widely used to clinically treat various diseases [71]. CPMs are manufactured according to the monographs of the *Pharmacopeia of the People’s Republic of China* using specific formulas stipulated by the Chinese law [25] and are considered clinically easier to formulate and safer to use than herbal decoctions owing to the laws and regulations [72]. The use of CPMP has been verified throughout the whole process from the beginning to the second half of the fracture, resulting in a significant increase in the osteoid thickness and mineral apposition rate and rapid stimulation of callus formation, osteoid formation, and maturity compared to that in the saline control group. Moreover, CPMP can be used as a treatment for delayed fracture healing or failure to achieve a normal fracture union [69]. In this study, seven types of CPMP (Diedashenggukeli, Guyulingjiaonang, Guzhecuoshangjiaonang, Huoxuezhitong, Jieguwan, Sanhuajiegusan, and Shangkejiegupian) were selected from the 33 CPMP types included in the *Pharmaceutical Dictionary* and the *Newly Edited National Chinese Traditional Patents*. Despite long-term use and therapeutic efficacy, there is a limited number of clinical trials investigating traditional mineral medicines, including pyritum, as remedies for fracture treatment. Moreover, there are no relevant systematic reviews or meta-analyses investigating the effect of CPMPs. This study serves as a reference for clinical use and the diverse research application of traditional mineral medicines, including pyritum.

In this study, CPMP was mostly used after reduction or fixation, such as open reduction and internal fixation or manual reduction and splinting. Although surgical treatment can effectively restore the anatomical position of the fracture and provide strong fixation, it has no obvious effect on the recovery of fracture healing. A previous study reported that postoperative side effects or complications may occur from delayed healing due to hematoma after the fracture [73]. To prevent complications during fracture healing, traditional medicine suggests that activating blood circulation and removing stagnation can promote the absorption and dissipation of blood, thereby removing harmful elements and creating favorable conditions for fracture healing [74]. The seven CPMPs included in this study are considered ideal selections of compounds because they have shown common efficacy in promoting blood circulation, dispelling blood stasis, reducing swelling, and relieving pain. Most of the individual components included in the seven CPMPs were blood-activating and stasis-dispelling medicinals. Excluding pyrite, the most used Tubiechong in CPMPs is an insect medicine that has the action of releasing blood stasis, facilitating blood circulation, eliminating lumps and masses, weaving tendons, and connecting bones in the *Chinese Pharmacopoeia*, and is included in more than 200 types of Chinese patent medicines [75]. In a previous study reporting TCM utilization patterns in patients with fractures [65], the single most prescribed herbs were Gusuibu and Xuduan, for strengthening tendons and bones, Yanhusuosuo, for moving qi and reducing pain, and for blood-quickening, stasis-transforming herbs including Danshen. In this study, the use of the mentioned herbs was confirmed. In TCM, theoretical grounds for the therapeutic effect of CPMP on fractures can be found, but additional research is needed to find the optimal combination of medicines including pyrite.

### 4.3. CPMP Safety

Regarding safety, four RCTs reported complications and side effects following the CPMP use. Postoperatively, the control groups received no treatment, and the treatment group received CPMP. One of the two studies reporting side effects revealed that there were no side effects in the CPMP group; however, rash occurred in the cefoxitin sodium + saline group postoperatively, which was considered to be related to cefoxitin sodium. In another report, fever and pain occurred in both the CPMP and control groups; however, there was no significant difference between the two groups. Regarding complications, three studies (one related to Jieguwan and two related to Shangkejiegupian) reported postoperative fracture dislocation, delayed union, functional disorder, infection, and deep vein thrombosis. Incidences of these complications were significantly lower in the CPMP group than in the control groups.

Fracture healing is a complex process involving various factors at the cellular and molecular levels and can lead to various complications, such as delayed healing or non-union healing pattern [63]. These complications are difficult to treat and result in a financial burden owing to the loss of productivity [66]. Accordingly, any strategy that helps reduce the healing time aids in the rapid resumption of work duties and daily activities by the patients, improvement of their medical outcomes, and reduction of their financial burden. As noted in our study, there were no serious side effects caused by CPMP, and the incidence of complications in the CPMP groups was lower than that in the control groups. Therefore, CPMP may be a relatively safe treatment strategy for fracture patients and can be used to reduce postoperative complications. Because adverse effects were not reported in most studies, the active observation of adverse effects and safety-related studies are needed for the safe clinical application of CPMP in the future.

### 4.4. Limitations and Suggestions for Further Studies

This study had several limitations. First, most of the included studies demonstrated high heterogeneity, publication bias, and regional bias. This could be attributed to the randomized-trial design of the included studies; however, there were no specific randomized trials for random sequence generation, allocation concealment, or outcome assessment blinding, and only 29 RCTs that were published in the same country were included. Second, seven types of CPMP were included for fracture treatment. High heterogeneity was observed owing to the different types of CPMPs, controls, and unequal treatment durations. Therefore, caution should be exercised when interpreting these results. Third, although CPMP had a statistically significant effect on treatment efficacy, pain reduction, and hematological change improvement in patients with fractures, most studies had a high risk of bias. In the future, high-quality clinical trials, with high validity and reliability and using a more rigorous methodology, are needed to evaluate the potential benefits of CPMP therapy for fractures.

Existing studies suggest that pyritum is useful for fracture treatment; however, no clinical trial has investigated this effect. However, clinical evidence could not be validated because pyritum was not used alone. Various clinical studies are required to verify the efficacy and safety of pyritum alone. Although limitations still persist in terms of the legal and clinical aspects of pyritum administration worldwide, this study could serve as a basis for the development of more treatment alternatives to patients with fractures, and for the design of future clinical studies.

## 5. Conclusions

In conclusion, compared with the results of the control group, CPMP was more effective in primary outcomes related to efficacy rate, such as total effective rate, callus growth rate, bone union, and edema disappearance time; secondary outcomes related to pain reduction, such as pain intensity and pain disappearance time; and blood test results, such as ESR, Hct, EA, and PA. In addition, CPMP did not cause serious side effects, and the incidence of complications was significantly less than that in the control group. This suggests that CPMP may be a useful and safe treatment for fractures. However, high-quality clinical trials with high validity and reliability are needed, as most of the included studies had a high risk of bias.

## Figures and Tables

**Table 1:** Study characteristics.

Study	Study Design	Sample Size (T/C)	Age (Years) (T/C)	Gender		Diagnostic Criteria	Fracture Type and Site	Operation Methods	Blinding Method
				M	F				
Lan 2009 [28]	RCT	40/40	44/45	52	28	N/A (postoperative)	Spinal (CV, TV, LV, TV and LV)	IF (screw-rod system/anterior plate)	N/A
He 2013 [29]	RCT	34/34	26–49	30	38	Diagnostic criteria for Colles fracture	Colles	MRS	N/A
Hong 2012 [30]	RCT	23/22	60–85/60–82	N/A	N/A	N/A (postoperative)	Colles	MRS	N/A
Zou 2013 [31]	RCT	30/30	20–29 10; 30~39 9: 40~49 11/20~29 11; 30~39 10: 40~49 9	34	26	Diagnostic criteria for fractures	Humerus, ulna or radius, ulna and radius	ORIF	N/A
Chi 2001 [32]	RCT	25/22	20–45	47	0	N/A (postoperative)	Tibiofibula	CR, IIN	N/A
Liu 2021 [33]	RCT	30/30	21~57 (38.92 ± 5.39)/ 22~58 (39.11 ± 5.43)	35	25	X-ray, CT	Tibiofibula	IIN	N/A
Niu 2020 [34]	RCT	100/100	21~76 (45.38 ± 7.24)/ 24~78 (46.12 ± 8.03)	132	68	Diagnostic criteria for fractures	Tibiofibula	ORIF	N/A
Xu 2018 [35]	RCT	30/30	33.2 ± 1.5/32.8 ± 1.8	38	22	Diagnostic criteria for fractures	Closed fractures around the knee joint	N/A	N/A
Zhou 2017 [36]	RCT	43/43	18~77 (56 ± 7.2)/ 18~78 (56.6 ± 7.1)	57	29	Diagnostic criteria for fractures	Tibiofibula	IIN	N/A
Tu 2018 [37]	RCT	47/47	36.02 ± 8.56/35.58 ± 8.13	59	35	N/A (postoperative)	Radius, femur, humerus, tibia	Reduction; IF; EF	N/A
Zhang 2018 [38]	RCT	60/60	49.3 ± 7.8/49.6 ± 9.4	89	31	Diagnostic criteria for long bone fractures	Humerus, ulna, radius, ulna and radius, femur, tibia, fibula, tibia and dibula	INN, EF; PSF; EF	N/A
Liu 1999 [39]	RCT	46/46	7–78	N/A	N/A	N/A (postoperative)	Humeral shaft, Humerus, ulna and radius, Colles, Metacarpal bone, pelvis, tibiofibula, medial and lateral ankles, metatarsal bone	MRS	N/A
Zhou 2000 [40]	RCT	62/40	34	64	38	N/A (postoperative)	Tibiofibula	Calcaneal traction	N/A
Chen 2015 [41]	RCT	44/44	45.18 ± 6.39	53	35	X-ray	Tibia, fibula, tibiofibular	ORIF	N/A
Gui 2019 [42]	RCT	43/43	44.51 ± 5.90/44.91 ± 5.82	52	34	Diagnostic criteria for pelvic fractures	Pelvic (tile C1~C3)	I: EF + NPWT; II: ORIF	N/A
He 2007 [43]	RCT	25/25	44/45	32	18	N/A (postoperative)	Tibia, tibiofibular, patella, multiple	IN/KWF	N/A
He 2019 [44]	RCT	41/41	39.8 ± 13.2/38.2 ± 12.8	44	38	X-ray	lower limb	RIF	N/A
He 2021 [45]	RCT	40/40	41.13 ± 5.62/41.49 ± 5.52	58	22	Imaging examination	Calcaneus	ORIF	N/A
Hua 2006 [46]	RCT	60/60	17–40 24; 41–60 22; 61–80 14/ 17–40 23; 41–60 22; 61–80 15	59	61	N/A (postoperative)	Ulna and radius, femur, patella, tibiofibular	ORIF; MRPF	N/A
Jin 2022 [47]	RCT	41/41	51.03 ± 6.18/50.90 ± 6.31	60	22	Diagnostic criteria for acute closed tibial fracture	Acute closed tibial fracture	IF	N/A
Li 2002 [48]	RCT	25/25	40.3/47.8	28	22	N/A (postoperative)	Femur, intertrochanteric, tibia, tibiofibular	N/A	N/A
Mei 2015 [49]	RCT	30/30	37.2 ± 3.8/38.3 ± 3.7	37	23	Diagnostic criteria for lower limb fracture	Multiple, patella, fibular, femur	IIN	N/A
Qi 2018 [50]	RCT	48/48	37.8 ± 10.3/36.5 ± 12.4	53	43	X-ray	Ankle	ORIF	N/A
Qiu 2020 [51]	RCT	40/39	49.62 ± 4.07/49.68 ± 4.11	0	79	N/A (postoperative)	Perimenopausal osteoporosis and unstable tibial plateau fracture	PPF	N/A
Shao 2013 [52]	RCT	40/40	40.2 ± 15.3/39.4 ± 14.7	49	31	X-ray	Humerus, tibia, femur	ORIF	N/A
Wang 2019 [53]	RCT	50/50	50.93 ± 4.26/50.28 ± 4.37	61	39	Diagnosed as ankle fracture	Closed ankle fracture	TF	N/A
Yan 2017 [54]	RCT	20/20	83.01 ± 1.24	18	22	MRI, CT, QCT, X-ray	Senior osteoporotic spinal compression (LV1~LV3)	PKP	N/A
Yang 2011 [55]	RCT	400/400	38.5	596	204	X-ray	Upper limb, lower limb, rib, spine, compound, pelvis	MREF; ORIF	N/A
Zhou 1999 [56]	RCT	104/104	46.5/44.5	145	63	N/A	Humerus, ulna and radius, femoral shaft, distal femur, tibiofibular, tibia	MN; EF	N/A

RCT, randomized controlled trial; T, treatment; C, control; M, male; F, female; N/A, not available; CT, computed tomography; MRI, magnetic resonance imaging; QCT, quantitative computed tomography; CV, cervical vertebra; TV, thoracic vertebra; LV, lumbar vertebra; IF, internal fixation; MRS, manual reduction and splint; ORIF, open reduction and internal fixation; CR, closed reduction; IIN, internal fixation and intramedullary nail; EF, external fixation; INN, interlocking intramedullary nail; PSF, plate and screw fixation; NPWT, negative pressure wound therapy; KWF, Kirshner wire fixation; RIF, reduction and internal fixation; MRPF, manual reduction and plate fixation; PPF, percutaneous plate fixation; MREF, manual reduction and external fixation; TF, tape fixation; PKP, percutaneous kyphoplasty; MN, medullary nailing.

**Table 2:** Characteristics of RCTs for the treatment of fractures.

Study	Treatments		Dosages/Duration		Outcomes		Adverse Effect
	Comparative Treatment	Treatment Included in Both Treatment and Control Groups	CPMP	Control	Main	Others	
Lan 2009 [28]	DDSG vs. none	OP	1 pk/t, 1/d, 7 d	N/A	ESR; PV; Hct; EA	WBV; WBRV; ED	N/A
He 2013 [29]	GYL vs. none	OP + VitD Ca + calcitriol	5 tb/t, 3/d, 4 wk	VitD Ca 2 tb/t, 1/d; calcitriol 1 tb/t, 3/d, 4 wk	EDT (d); PDT; BUE (d); CGR; TER	N/A	N/A
Hong 2012 [30]	GYL vs. none	OP	5 tb/t, 3/d, 4 wk	N/A	BUE (d); CGR; TER	N/A	N/A
Zou 2013 [31]	GYL vs. none	OP	6 tb/t, 3/d, 8 wk	N/A	VAS	Edema; FL; CG	N/A
Chi 2001 [32]	GZCS vs. none	OP	3 tb/t, 3/d, 8–12 wk	N/A	CGR	BUE (mo); EDT (wk)	N/A
Liu 2021 [33]	HXZT vs. none	OP + mezlocillin injection	1.0 g/t, 3/d, 5 d	Mezlocillin injection 2.0 g, 2/d, 3~5 d	TER; PDT	ROM; PICP; BGP; β-CTX; Fb; PLT	N/A
Niu 2020 [34]	HXZT vs. none	OP	1.0 g/t, 3/d, 7 d	N/A	TER; VAS	BGP; BMP-2; Calcitonin; PICP; D-D; PT; Fb; APTT	None
Xu 2018 [35]	HXZT vs. none	OP + sodium chloride injection + cefazolin sodium pentahydrate	1.0 g/t, 3/d, 7 d	0.9% sodium chloride injection 1000 mL + cefazolin sodium pentahydrate 2.0 g, 1–3 d	TER; VAS; EDT (d)	BUE (wk)	None
Zhou 2017 [36]	HXZT vs. none	OP + cefoxitin sodium + saline	1.0 g/t, 3/d, 5 d	Cefoxitin sodium for injection 2.0 g + 100 mL saline 1/d, 35 d	TER; EDT (d); PDT	NRS	T: None; C: rash (1)
Tu 2018 [37]	JGW vs. none	OP	1 tb/t, 2/d, 4 wk	N/A	PDT; TER	CRP; TNF-α; IL-6; BUE (wk); EDT (wk)	None
Zhang 2018 [38]	JGW vs. none	OP	1 tb/t, 2/d, 6 wk	N/A	TER; BUE (d); PDT; EDT (d)	BMP-7; LEP	T: FM (1); DU (1); DF (1); LD (2)/C: FM (3); DU (5); Inf (2); DF (3); LD (3)
Liu 1999 [39]	SHJG vs. none	OP	1 pk (5 g)/t, ~3 yr: 1/3 pk, 3 yr~: 1/2 pk, 2/d, 14 d	Antd 2 mL; CAP tb 2 tb, 3/d; OC tb 100 mg, 3/d; Vit AD, 1 tb, 3/d; 10% Glu 50 mL, β-SA 25 mg, 1/d; 20%, Man 250 mL, 1/d, 7 d	CGR	PER; EER	N/A
Zhou 2000 [40]	SHJG vs. none	Calcaneal traction + Vit C + OC	1 pk (5 g)/t, 2/d, 14 d	Vit C, OC tb 4 tb, 3/d	CGR	N/A	N/A
Chen 2015 [41]	SKJG vs. none	OP	10–14 yr: 3 tb/t; 14~: 4 tb/t, 3/d, 8 wk	N/A	VAS; EDT (d); TER	LOS; CG; BUE (wk)	N/A
Gui 2019 [42]	SKJG vs. Antibiotics + HS	OP	4 tb/t, 3/d, 3 mo	Antibiotics (6–7 d); HS (2 wk)	ESR	Fb; VR; WBV	N/A
He 2007 [43]	SKJG vs. none	OP	4 tb/t, 3/d	N/A	ESR; Hct; PV; EA	Fb; ED; WBRV	N/A
He 2019 [44]	SKJG vs. none	OP	4 tb/t, 3/d, 30 d	N/A	TER; ESR; Hct; PV; EA	CRP; ALP; CF; WBRV; ED	N/A
He 2021 [45]	SKJG vs. none	OP	4 tb/t, 3/d, 12 wk	N/A	BUE (d); TER	CM	T: DVT (1)/C: Inf (1), DVT (3), hem (2)
Hua 2006 [46]	SKJG vs. none	OP	4 tb/t, 3/d, 6 wk	N/A	TER	N/A	N/A
Jin 2022 [47]	SKJG vs. none	OP + HS	3/d, 1.32 g/t, 12 wk	HS Injection, 1/d, 0.4 mL/t, 6 d	VAS; TER	BUR	N/A
Li 2002 [48]	SKJG vs. OP	-	4 tb/t, 7 d	N/A	ESR; PV; Hct; EA	WBRV; ED; CF	N/A
Mei 2015 [49]	SKJG vs. none	SA	4 tb/t; 3/d, 7 d	SA 30 mg + 10% Glu 1/d, 7 d	TER; PV; ESR	Fb	N/A
Qi 2018 [50]	SKJG vs. none	OP	4 tb/t, 3/d, 4 wk	N/A	TER	CM	N/A
Qiu 2020 [51]	SKJG vs. none	OP	3/d, 4 tb/t, 6 mo	N/A	PDT; EDT (d); BUE (d)	BALP; Ca; BD; CG; Cal	Adverse effects T: 25/40 (62.50%), fever 23, ache 20; C: 24/40 (61.54%), fever 23, ache 19 Complications T:5/40 (12.50%), DU 2, LIF 3 C: 10/39 (25.64%), DU 5, LIF5
Shao 2013 [52]	SKJG vs. none	OP	4 tb/t, 3/d, 30 d	N/A	TER	CG; BUE (wk)	None
Wang 2019 [53]	SKJG vs. none	OP + DSI	3/d, 4 tb/t, 4 d	DSI 50 mg/t, 2–3/d	TER; EDT (d); VAS	CM; LOS	None
Yan 2017 [54]	SKJG vs. none	PKP	4 tb/t, 4/d, 8 wk	N/A	VAS	ODI; VBH	N/A
Yang 2011 [55]	SKJG vs. benorilate	OP	2 tb/t, 3/d (10 yr); 4 tb/t, 3/d (Ad)	benorilate 1–2 tb/t, 3/d, 60 d	CGR	SD	N/A
Zhou 1999 [56]	SKJG vs. none	OP	4 tb/t, 3/d, 30 d	N/A	CGR	EDR; BUE (wk)	N/A

N/A, not available; CPMP, Chinese patent medicine containing pyritum; DDSG, Diedashenggukeli; GYL, Guyulingjiaonang; GZCS, Guzhecuoshangjiaonang; HXZT, Huoxuezhitong; JGW, Jieguwan; SHJG, Sanhuajiegusan; SKJG, Shangkejiegupian; OP, operation; pk/t, pack/time; g/t, gram/time; tb/t, tablet/time; d, day; wk, week; mo, month; yr, year; Ad, adult; ESR, erythrocyte sedimentation rate; PV, plasma viscosity; Hct, hematocrit; WBV, whole blood viscosity; WBRV, whole blood reducing viscosity; EA, DSIerythrocyte aggregation; ED, erythrocyte deformability; EDT, edema disappearance time; PDT, pain disappearance time; BUE, bone union evaluation; BUR, bone union rate; CG, callus growth; CGR, callus growth rate; TER, total effective rate; VAS, visual analogue scales; FL, fracture line; ROM, range of motion rom; PICP, procollagen type I carboxy-terminal; BGP, bone Gla protein; β-CTX, β-isomerized C-terminal telopeptides; Fb, fibrinogen; PLT, platelet; BMP-2, bone morphogenetic protein 2; D-D, D-dimer; PT, prothrombin time; APTT, activated part thromboplastin time; NRS, numerical rating scale; CRP, C-reactive protein; TNF-α, tumor necrosis factor-α; IL-6, interleukin-6; BMP-7, bone morphogenetic protein 7; LEP, leptin; PER, pain effective rate; EER, edema effective rate; LOS, length of stay; VR, viscosity ratio; ALP, alkaline phosphatase; CF, coagulation factor; CM, calcaneal morphology; BALP, bone alkaline phosphatase; Ca, calcium; Cal, calcitonin; BD, bone density; LIF, loosening internal fixture; ODI, Oswestry disability index; VBH, vertebral body height; SD, symptom disappearance rate; EDR, edema disappearance rate; Antd, antonidine; CAP, compound aminopyrine phenacetin; OC, oyster shell calcium; Glu, glucose; SA, sodium aescinate; Man, mannitol; VitD Ca, vitamin D calcium; HS, heparin sodium injection; Vit C, vitamin C; DSI, diclofenac sodium injection; FM: fracture displacement; DU: delayed union; DF, dysfunction; LD, limb deformity; Inf, infection; DVT, deep vein thrombosis; hem, hematoma.

**Table 3:** Summary of the evidence and effects of CPMP interventions for fractures.

Characteristic	No. of Studies
Main Varieties	
Diedashenggu keli (granules)	1
Guyuling jiaonang (capsule)	3
Guzhecuoshang jiaonang (capsule)	1
Huoxuezhitong jiaonang (capsule)	4
Jiegu wan (pill)	2
Sanhuajiegu san (powder)	2
Shangkejiegu pian (pill)	16
Outcomes	
Total effective rate	17 (16+, 1−)
Callus growth rate	7 (7+)
Evaluation of bone union	5 (5+)
Edema disappearance time	7 (7+)
VAS pain score	7 (6+, 1−)
Pain disappearance time	6 (6+)
Erythrocyte sedimentation rate	6 (5+, 1−)
Hematocrit	4 (4+)
Erythrocyte aggregation	4 (3+, 1−)
Plasma viscosity	5 (4+, 1−)

+ overall beneficial effect; − no effect. CPMP, Chinese patent medicine containing pyritum.

**Table 4:** Overview of CPMP ingredients for fractures.

Main Varieties	Drug Composition (Chinese Pinyin)	Approval No. of SFDA (State Food and Drug Administration in China)	Prescription Functions (TCM Patterns)
Diedashenggu keli (granules)	Caulis Premnae Fulvae (Zhangu), Sarcandrae Herba (Zhongjiefeng), Pyritum (Zirantong), Salviae Miltiorrhizae Radix Et Rhizoma (Danshen), Corydalis Rhizoma (Yanhusuo), Achyranthis Bidentatae Radix (Niuxi), Eucommiae Cortex (Duzhong), Dextrin	Z20025338	Improve blood circulation and disperse stasis, reduce swelling and alleviate pain, and strengthen muscles and bones.
Guyuling jiaonang (capsule)	Notoginseng Radix Et Rhizoma (Sanqi), Draconis Sanguis (Xuejie), Carthami Flos (Honghua), Angelicae Sinensis Radix (Danggui), Rhizoma Chuanxiong (Chuanxiong), Paeoniae Radix Rubra (Chishao), Olibanum (Ruxiang), Commiphora Myrrha (Moyao), Rhei Radix Et Rhizoma (Dahuang), Dipsaci Radix (Xudan), Drynariae Rhizoma (Gusuibu), Acanthopanacis Cortex (Wujiapi), Rehmanniae Radix Praeparata (Shudihuang), Pyritum (Zirantong), Paeoniae Radix Alba (Baishao), Sodium tetraborate (Pengsha)	Z20025015	Improve blood circulation and disperse stasis, reduce swelling and alleviate pain, and strengthen muscles and bones. Used for fractures and osteoporosis.
Guzhecuoshang jiaonang (capsule)	Pig’s bone (Zhugu), Semen Cucumis Sativi (Huangguazi), Eupolyphaga Steleophaga (Tubiechong), Pyritum (Zirantong), Olibanum (Ruxiang), Commiphora Myrrha (Moyao), Draconis Sanguis (Xuejie), Carthami Flos (Honghua), Rhei Radix Et Rhizoma (Dahuang), Angelicae Sinensis Radix (Danggui)	Z20053201	Stimulate the circulation of the blood and cause the muscles and joints to relax, join bone, and relieve pain. Used for injuries from falls, reducing swelling and dissipating blood stasis, lumbar swelling, upper limb pain, etc.
Huoxuezhitong jiaonang (capsule)	Angelicae Sinensis Radix (Danggui), Notoginseng Radix Et Rhizome (Sanqi), Olibanum (Ruxiang), Borneolum (Bingpian), Eupolyphaga Steleophaga (Tubiechong),Pyritum (Zirantong)	Z10920002	Improve blood circulation and disperse stasis, reduce swelling, and alleviate pain. Used for injuries from falls, reducing swelling, and dissipating blood stasis.
Jiegu wan (pill)	Melo Semen (Tianguazi), Eupolyphaga Steleophaga (Tubiechong), Pheretima (Dilong), Cinnamomi Ramulus (Guizi), Curcumae Radix (Yujin), Drynariae Rhizoma (Gusuibu), Dipsaci Radix (Xudan), Pyritum (Zirantong), Semen Strychni Pulveratum (Maqianzifen)	Z22025709	Improve blood circulation and disperse stasis, reduce swelling, and alleviate pain. Used for injuries from falls, purplish swelling and pain, lumbar swelling, upper limb pain, fracture, and blood stasis and pain.
Sanhuajiegu san (powder)	Notoginseng Radix Et Rhizome (Sanqi), Croci Stigma(Xihonghua), Strychni Semen (Maqianzi), Cinnamomum cassia (Guipi), Aquilariae Lignum Resinatum (Chenxiang), Angelicae sinensis radix (Danggui), Pheretima (Dilong), Achyranthis Bidentatae Radix (Niuxi), Borneolum (Bingpian), Aucklandiae Radix (Muxiang), Rhizoma chuanxiong (Chuanxiong), Eupol-yphaga Steleophaga (Tubiechong), Dipsaci Radix (Xudan), Drynariae Rhizoma (Gusuibu), Draconis Sanguis (Xuejie), Rhei Radix Et Rhizoma (Dahuang), Pyritum (Zirantong), Angelica Dahuricae Radix powder (Baizhifen)	Z10950013	Improve blood circulation, disperse stasis, reduce swelling, alleviate pain, and reunite bone. Used for fracture and tendon injury, blood stasis, and pain.
Shangkejiegu pian (pill)	Carthami Flos (Honghua), Eupolyphaga Steleophaga (Tubiechong), Cinnabaris (Zhusha), Semen Strychni Pulv-eratum (Maqianzifen), Commiphora Myrrha (Moyao), Notoginseng Radix Et Rhizome (Sanqi), Star Fish (Haix-ing), Chicken bone (Jigu), Borneolum (Bingpian), Pyritu-m (Zirantong), Olibanum (Ruxiang), Melo Semen (Tianguazi)	Z21021461	Improve blood circulation and disperse stasis, reduce swelling and alleviate pain, soothe the sinews, and strengthen the bones. Used for injuries from falls, purplish swelling and pain, lumbar swelling, upper limb pain, fracture, and blood stasis and pain. Patients with fractures should be treated with reduction before use.

CPMP, Chinese patent medicine containing pyritum.

**Table 5:** Methodological quality of included studies according to the tool Risk of Bias 2.0.

Study	D1	D2	D3	D4	D5	Overall
Chen 2015 [18]	L	Sc	L	Sc	Sc	Sc
Chi 2001 [9]	L	Sc	L	Sc	Sc	Sc
Gui 2019 [19]	L	Sc	L	Sc	Sc	Sc
He 2007 [20]	L	Sc	L	Sc	Sc	Sc
He 2013 [6]	Sc	Sc	L	Sc	Sc	Sc
He 2019 [21]	Sc	Sc	L	Sc	Sc	Sc
He 2021 [22]	L	Sc	L	Sc	Sc	Sc
Hong 2012 [7]	H	Sc	L	Sc	Sc	H
Hua 2006 [23]	H	Sc	L	Sc	Sc	H
Jin 2022 [24]	L	Sc	L	Sc	Sc	Sc
Lan 2009 [5]	Sc	Sc	L	Sc	Sc	Sc
Li 2002 [25]	Sc	Sc	H	Sc	Sc	H
Liu 1999 [16]	Sc	Sc	L	Sc	Sc	Sc
Liu 2021 [10]	Sc	Sc	L	Sc	Sc	Sc
Mei 2015 [26]	Sc	Sc	L	Sc	Sc	Sc
Niu 2020 [11]	L	Sc	L	Sc	Sc	Sc
Qi 2018 [27]	L	Sc	L	Sc	Sc	Sc
Qiu 2020 [28]	L	Sc	L	Sc	Sc	Sc
Shao 2013 [29]	Sc	Sc	L	Sc	Sc	Sc
Tu 2018 [14]	Sc	Sc	L	Sc	Sc	Sc
Wang 2019 [30]	L	Sc	L	Sc	Sc	Sc
Xu 2018 [12]	L	Sc	L	Sc	Sc	Sc
Yan 2017 [31]	L	Sc	L	Sc	Sc	Sc
Yang 2011 [32]	H	Sc	L	Sc	Sc	Sc
Zhang 2018 [15]	Sc	Sc	L	Sc	Sc	Sc
Zhou 1999 [33]	Sc	Sc	L	Sc	Sc	Sc
Zhou 2000 [7]	H	Sc	L	Sc	Sc	H
Zhou 2017 [13]	L	Sc	L	Sc	Sc	Sc
Zou 2013 [8]	Sc	Sc	L	Sc	Sc	Sc

D1–D5: 5 domain criteria; D1, bias arising from the randomization process; D2, bias due to deviations from intended interventions; D3, bias due to missing outcome data; D4, bias in the measurement of the outcome; D5, bias in the selection of the reported results.; H: high risk of bias; L: low risk of bias; Sc: some concerns.

**Table 6:** Summary of study findings in this meta-analysis.

Intervention	Outcomes	Number of Participants (Studies)	Anticipated Absolute Effects (95% CI)	Quality of the Evidence (GRADE)
Comparison of CPMP and control (no CPMP or WM) for fracture	Total efficacy rate	1539 (17)	197 fewer per 1000 (from 258 fewer to 143 fewer)	⨁⨁⨁◯ Moderate *
	Callus growth rate	1362 (7)	192 fewer per 1000 (275 fewer to 124 fewer)	⨁⨁⨁◯ Moderate ^†^
	Evaluation of bone union	392 (5)	SMD 1.28 lower (1.94 lower to 0.63 lower)	⨁⨁◯◯ Low * ^†^
	Edema disappearance time	601 (7)	SMD 1.23 lower (1.59 lower to 0.88 lower)	⨁⨁◯◯ Low * ^†^
	VAS	650 (7)	SMD 1.62 lower (2.76 lower to 0.49 lower)	⨁⨁◯◯ Low * ^†^
	Pain disappearance time	507 (6)	SMD 1.72 lower (2.59 lower to 0.85 lower)	⨁⨁◯◯ Low * ^†^
	ESR	408 (6)	SMD 1.07 lower (1.73 lower to 0.4 lower)	⨁⨁◯◯ Low * ^†^
	Hct	262 (4)	SMD 0.72 lower (1.08 lower to 0.36 lower)	⨁⨁⨁◯ Moderate *
	EA	262 (4)	SMD 2.53 lower (4.75 lower to 0.31 lower)	⨁⨁◯◯ Low * ^†^
	PV	322 (5)	SMD 0.93 lower (1.77 lower to 0.09 lower)	⨁⨁◯◯ Low * ^†^

CI, confidence interval; CPMP, Chinese patent medicine containing pyritum; WM, Western medicine; SMD, standardized mean difference; ESR erythrocyte sedimentation rate; Hct, hematocrit; EA, erythrocyte aggregation; PV, plasma viscosity. * Substantial concerns of publication bias; ^†^ The confidence intervals are less overlapping.

## Data Availability

The original contributions presented in the study are included in the article. Further inquiries can be directed to the corresponding authors.

## References

[1] Buccino F., Zagra L., Savadori P., Galluzzo A., Colombo C., Grossi G., Banfi G., Vergani L.M. (2021). Mapping local mechanical properties of human healthy and osteoporotic femoral heads. Materialia.

[2] Buccino F., Martinoia G., Vergani L.M. (2021). Torsion—Resistant Structures: A Nature Addressed Solution. Materials.

[3] Kasiri S., Taylor D. (2008). A critical distance study of stress concentrations in bone. J. Biomech..

[4] Fazzalari N.L. (2011). Bone fracture and bone fracture repair. Osteoporos. Int..

[5] Vestergaard P., Krogh K., Rejnmark L., Mosekilde L. (1998). Fracture rates and risk factors for fractures in patients with spinal cord injury. Spinal Cord..

[6] GBD 2019 Fracture Collaborators (2021). Global, regional, and national burden of bone fractures in 204 countries and territories, 1990-2019: A systematic analysis from the Global Burden of Disease Study 2019. Lancet Healthy Longev..

[7] Polinder S., Haagsma J., Panneman M., Scholten A., Brugmans M., Van Beeck E. (2016). The economic burden of injury: Health care and productivity costs of injuries in the Netherlands. Accid Anal. Prev..

[8] Borgström F., Karlsson L., Ortsäter G., Norton N., Halbout P., Cooper C., Lorentzon M., McCloskey E.V., Harvey N.C., Javaid M.K. (2020). Fragility fractures in Europe: Burden, management and opportunities. Arch Osteoporos..

[9] Tatangelo G., Watts J., Lim K., Connaughton C., Abimanyi-Ochom J., Borgström F., Nicholson G.C., Shore-Lorenti C., Stuart A.L., Iuliano-Burns S. (2019). The cost of osteoporosis, osteopenia, and associated fractures in Australia in 2017. J. Bone Miner Res..

[10] Loi F., Córdova L.A., Pajarinen J., Lin T.H., Yao Z., Goodman S.B. (2016). Inflammation, fracture and bone repair. Bone.

[11] Endo K., Sairyo K., Komatsubara S., Sasa T., Egawa H., Yonekura D., Adachi K., Ogawa T., Murakami R.-I., Yasui N. (2002). Cyclooxygenase-2 inhibitor inhibits the fracture healing. J. Physiol. Anthropol. Appl. Human Sci..

[12] Chiodini I., Bolland M.J. (2018). Calcium supplementation in osteoporosis: Useful or harmful?. Eur. J. Endocrinol..

[13] Kennel K.A., Drake M.T. (2009). Adverse effects of bisphosphonates: Implications for osteoporosis management. Mayo Clin. Proc..

[14] Song K., Yao Y., Rong Z., Shen Y., Zheng M., Jiang Q. (2016). The preoperative incidence of deep vein thrombosis (DVT) and its correlation with postoperative DVT in patients undergoing elective surgery for femoral neck fractures. Arch Orthop. Trauma. Surg..

[15] Calori G.M., Albisetti W., Agus A., Iori S., Tagliabue L. (2007). Risk factors contributing to fracture non-unions. Injury.

[16] Zhang D., Potty A., Vyas P., Lane J. (2014). The role of recombinant PTH in human fracture healing: A systematic review. J. Orthop. Trauma..

[17] Babu S., Sandiford N.A., Vrahas M. (2015). Use of teriparatide to improve fracture healing: What is the evidence?. World J. Orthop..

[18] Son Y.J. (2019). Studies for Bone Formation of Herbal Medicines on Femoral Fracture Model in Rat. https://scienceon.kisti.re.kr/srch/selectPORSrchReport.do?cn=TRKO201900019965.

[19] Sung S.H. (2012). Botanical Drug Product. Molecular Biology Newsletter. https://www.ksmcb.or.kr/file/webzine/2012_10_03.pdf.

[20] Hwang J.H., Ahn J.H., Kim J.T., Ahn S.H., Kim K.H., Cho H.S., Lee S.D., Kim E.J., Kim K.S. (2009). Effects of administration of Pyritum on activation of osteoblast cells in human body and on tibia bone fracture in mice. J. Korean Acupunct Moxibustion Soc..

[21] Shin K.M., Jung C.Y., Hwang M.S., Lee S.D., Kim K.H., Kim K.S. (2009). Effects of administration of Pyritum on fracture healing in mice. J. Korean Acupunct Moxibustion Soc..

[22] Wang L.L., Zuo R.T., Chen S.Q. (2017). Analysis on therapeutic effects and adverse reactions of Chinese patent drug containing mineral medicine. Chin. Med. Pharm..

[23] Liu K., Zhang Y., Song X. (2021). Effectiveness of Chinese patent medicine in the treatment of influenza: A protocol for systematic review and meta-analysis. Medicine.

[24] Hu Z.C. (2020). A Network Meta-analysis of the Effect of Five Oral Chinese Herbal Prescriptions on Limb Fractures. Master’s Thesis.

[25] Choi S.H., Nam E.Y., Hwang J.H. (2022). Therapeutic efficacy of Chinese patent medicine containing pyrite for fractures: A protocol for systematic review and meta-analysis. Medicine.

[26] Page M.J., McKenzie J.E., Bossuyt P.M., Boutron I., Hoffmann T.C., Mulrow C.D., Shamseer L., Tetzlaff J.M., Akl E.A., Brennan S.E. (2021). The PRISMA 2020 statement: An updated guideline for reporting systematic reviews. BMJ.

[27] Xu X.T. (2012). Practice of Orthopaedics.

[28] Lan C.G., Tang Y.J., Lu M.A., Xie K.G., Wei W. (2009). The effect of Diedashenggu granule on the hemorheology of patients after internal fixation of spine fracture. J. Cervicodynia Lumbodynia..

[29] He J.Y. (2013). Clinical observation of 34 cases of Colles fracture treated by Guyuling capsule. New Chin. Med..

[30] Hong H.C., Liu X., Huang M.J., Zhong W.L., Ceng G.X., Wu C.J. (2012). Clinical observation of the treatment of senile distal radius fracture with manual reduction and small splint fixation combined with Guyuling capsule. Guide Chin. Med..

[31] Zou Z.K. (2013). The Clinical Observation of the Treatment of Upper Limb Fracture by Open Reduction and Internal Fixation with GuYuLing Capsule. Master’s Thesis.

[32] Chi L.T., Pei F.X., Yang T.F., Tu Z.Q., Li J., Ning N. (2001). Clinical study of Guzhe Chuoshang capsule in promoting fracture healing. West China Med. J..

[33] Liu H.F., Wang Z.P. (2021). Effect of Huoxue Zhitong capsule on postoperative bone metabolism in patients with tibiofibular fracture. Chin. J. Thromb. Hemost..

[34] Niu X.G., Zhang M.L. (2020). Effect of Huoxuezhitong capsule on bone metabolism and curative effect aft-er tibia and fibula fracture. World J. Integr. Trad West Med..

[35] Xu B., Jia L., Zhang H. (2018). Therapeutic effect of Huoxue Zhitong capsule on postoperative recovery of closed fractures around the knee joint. J. Shaanxi Univ. Chin. Med..

[36] Zhou J.E. (2017). Effect of Huoxue Zhitong capsules for postoperative pain and swelling in patients with fracture of tibia and fibula. New Chin. Med..

[37] Tu H.H., Li Y.X. (2018). Clinical effect of Jiegu Pills treating patients with limb fracture. Chin. Mod. Med..

[38] Zhang Y.F., Zhong Q. (2018). Effect of Jiegu pill on morphogenetic Protein-7 and serum leptin in patients with long tubular bone fracture. Mod. J. Integr. Trad Chin. West Med..

[39] Liu Y.B. (1999). Clinical observation of Sanhua Jiegu powder in treating fracture. Hebei J. TCM Publ..

[40] Zhou Q.Y., Lu G.L., Yu M., Wu Y.J. (2000). Treatment of tibiofibular fracture with Sanhua Jiegu powder and calcaneal traction. Chin. J. Trad Med. Traum. Orthop..

[41] Chen H.B., Yang S.W. (2015). Clinical observation on 44 cases of tibiofibular fracture treated by Shang-ke Jiegu tablet combined with western medicine. J. New Chin. Med..

[42] Gui J.J., Liang Y., Dai Y.Y., Qi A.L. (2019). Effect of Shangke Jiegu tablet on syndrome of blood stasis and stagnation after pelvic fracture. ZH J. J. Trauma..

[43] He M.L., Xiao Z.M., Chen A.M. (2007). Effect of Shangke Jiegu tablet on hemorheology of patients with lower limb fracture after internal fixation. J. Guangxi Med. Univ..

[44] He H.L., Qiu D.Y., Lian J. (2019). Clinical observation of Shangke Jiegu tablet on patients with lower Li-mb fracture after internal fixation. Heilongjiang Med. J..

[45] He C.F., Yu L.C., Zhao L.F., Ren G.W. (2021). Observation on the effect of open reduction and internal fixation combined with Shangke Jiegu Tablet in the treatment of early intra-articular calcaneal fractures. ZH J. J. Trauma..

[46] Hua Y.X. (2006). 60 cases of fracture treated with Shangke Jiegu tablet. Henan Trad. Chin. Med..

[47] Jin X.J., Zhan X.L., You X.B. (2022). Clinical study on Shangke Jiegu pills combined with internal fixation with locking plate for tibial fracture. New Chin. Med..

[48] Li G.H., Chen C., Xia R.Y. (2002). The hemorheological effect of Shangke (department of traumatology) bone-knitting tablets in patients with lower limb fracture. Herald. Med..

[49] Mei S.T. (2015). Effect of Shangke Jiegu tablet on hemorheology of patients with lower limb fracture after internal fixation. J. New Chin. Med..

[50] Qi W.L., Dong S.Z. (2018). Effect of Shangke Jiegu tablet on improving the healing of ankle fracture after operation. Chin. J. Rural Med. Pharm..

[51] Qiu Y.Y., Xie Y., Chen C.Y., Xie Q.Y., Lin Z.X., Ye J.J. (2020). Clinical efficacy of Shangke Jiegu tablet combined with zoledronic acid in the treatment of perimenopausal osteoporosis and unstable tibial plateau fracture. Chin. J. Gerontol..

[52] Shao R.H., Zhu X.M. (2013). Treatment of 40 cases of fracture with Shangke Jiegu tablet. China Pharm..

[53] Wang X.X., Yang J. (2019). Clinical observation of Shangke Jiegu Tablets combined with diclofenac in treatment of early swelling of closed ankle and foot fractures. Drugs Clin..

[54] Yan J.T., Yan C.H., Zhao S.H., Yang Y.P., Wu B., Xiao Q., Feng K., Dong Q.Q. (2017). Observation on clinical effect of PKP and Chinese patent medicine in treating senile patients with old osteoporotic vertebral compression fractures. World Chin. Med..

[55] Yang G.Q., Ran Q.M., Yang R.X., Chen Z.L., Hu Y.L. (2011). Treatment of 400 cases of traumatic fracture with Shangke Jiegu tablet. Chin. Med. Mod. Distance Educ. China.

[56] Zhou J.S., Li Y.S., Yuan C., Wang Z.Q., Cheng C. (1999). Clinical observation on the treatment of traumatic fracture with Shangke Jiegu tablet. Chin. J. Traumatol..

[57] Sterne J.A.C., Savović J., Page M.J., Elbers R.G., Blencowe N.S., Boutron I., Cates C.J., Cheng H.Y., Corbett M.S., Eldridge S.M. (2019). RoB 2: A revised tool for assessing risk of bias in randomised trials. BMJ.

[58] Biswas L., Chen J., De Angelis J., Singh A., Owen-Woods C., Ding Z., Pujol J.M., Kumar N., Zeng F., Ramasamy S.K. (2023). Lymphatic vessels in bone support regeneration after injury. Cell.

[59] Owen-Woods C., Kusumbe A. (2022). Fundamentals of bone vasculature: Specialization, interactions and functions. Semin. Cell Dev. Biol..

[60] Khajuria D.K., Reider I., Kamal F., Norbury C.C., Elbarbary R.A. (2023). Distinct defects in early innate and late adaptive immune responses typify impaired fracture healing in diet-induced obesity. Front. Immunol..

[61] Khajuria D.K., Nowak I., Leung M., Karuppagounder V., Imamura Y., Norbury C.C., Kamal F., Elbarbary R.A. (2023). Transcript shortening via alternative polyadenylation promotes gene expression during fracture healing. Bone Res..

[62] Tsukasaki M., Takayanagi H. (2019). Osteoimmunology: Evolving concepts in bone-immune interactions in health and disease. Nat. Rev. Immunol..

[63] Marsh D. (1998). Concepts of fracture union, delayed union, and nonunion. Clin. Orthop. Relat. Res..

[64] Hak D.J., Fitzpatrick D., Bishop J.A., Marsh J.L., Tilp S., Schnettler R., Simpson H., Alt V. (2014). Delayed union and nonunions: Epidemiology, clinical issues, and financial aspects. Injury.

[65] Tseng C.Y., Huang C.W., Huang H.C., Tseng W.C. (2018). Utilization pattern of traditional Chinese medicine among fracture patients: A Taiwan hospital-based cross-sectional study. Evid. Based Complement Altern. Med..

[66] Yuan Z. (2022). Assessment of the safety and efficacy of the Chinese herbal formula (CHF) in fracture treatment. Proceedings of the 12th International Conference on Biomedical Engineering and Technology (ICBET 2022).

[67] Chalmers J., Gray D.H., Rush J. (1975). Observations on the induction of bone in soft tissues. J. Bone Joint. Surg. Br..

[68] Mohr R., Scherer P.R. (1976). Accelerated fracture healing. J. Am. Podiatr. Med. Assoc..

[69] Keum D.H., Kim S.S. (2002). Healing effect of pyrite on Tibia Fractured Rats. J. Orient. Rehabil. Med..

[70] Kim S.O., Park M.E. (2015). Standardization studies for the oriental mineral medicine. Econ. Environ. Geol..

[71] Zhang J.L. (2018). Chinese Patent Medicines.

[72] State Pharmacopoeia Commission of the PRC (2005). Pharmacopoeia of the People’s Republic of China.

[73] Bibbo C., Lin S.S., Beam H.A., Behrens F.F. (2001). Complications of ankle fractures in diabetic patients. Orthop. Clin. N. Am..

[74] Xue C., Pan W., Lu X., Guo J., Xu G., Sheng Y., Yuan G., Zhao N., Sun J., Guo X. (2021). Effects of compound deer bone extract on osteoporosis model mice and intestinal microflora. J. Food Biochem..

[75] Fu X., Shao B.-H., Wei X., Wang H.-H., Chen X., Zhao T.-T., Wang C.-M. (2022). Tubiechong: A review on ethnomedicinal uses, bioactive chemical constituents and pharmacological activities. J. Ethnopharmacol..

